# The value of *CEP55* gene as a diagnostic biomarker and independent prognostic factor in LUAD and LUSC

**DOI:** 10.1371/journal.pone.0233283

**Published:** 2020-05-21

**Authors:** Linhai Fu, Haiyong Wang, Desheng Wei, Bin Wang, Chu Zhang, Ting Zhu, Zhifeng Ma, Zhupeng Li, Yuanlin Wu, Guangmao Yu

**Affiliations:** The Department of Thoracic and Cardiovascular Surgery, Shaoxing People’s Hospital, Shaoxing Hospital, Zhejiang University School of Medicine, Hangzhou, Zhejiang, Republic of China; Shantou University Medical College, CHINA

## Abstract

**Objective:**

To investigate the value of *CEP55* as a diagnostic marker and independent prognostic factor in lung adenocarcinoma (LUAD) and squamous cell carcinoma (LUSC), and to analyze its co-expression genes and related signaling pathways.

**Methods:**

TCGA database and GEO database were used to analyze the expression of *CEP55* in LUAD and LUSC compared with normal tissues. The co-expression genes of *CEP55* in LUAD and LUSC were excavated by cBioPortal and enriched by KEGG and GO. Establishing Receiver operating characteristic (ROC) curve to evaluate the value of *CEP55* as a diagnostic and prognostic factor. The association between *CEP55* expression and the clinicopathological features was evaluated using χ2 tests. ROC curves for diagnosis and prognosis detection were constructed. Prognostic values were analyzed by univariate and multivariate Cox regression models.

**Results:**

Compared with normal lung tissues, *CEP55* expression was significantly upregulated in both LUAD and LUSC. ROC curve analysis showed that CEP55 could be used as an effective diagnostic target for LUAD (AUC = 0.969) and LUSC (AUC = 0.994). When *CEP55* gene was selected as an independent prognostic factor, high expression of *CEP55* was more disadvantageous to OS and RFS of LUAD patients (P<0.05), but no significant difference was found in LUSC patients (P>0.05). The number of co-expression genes of *CEP55* in LUAD is more than that in LUSC, and is related to cell cycle, DNA replication and P53 signaling pathway.

**Conclusion:**

*CEP55* can be used as a diagnostic marker for LUAD and LUSC, but only as an independent prognostic factor for LUAD rather than LUSC.

## Introduction

Lung cancer is one of the most common types of cancer. In 2018, lung cancer accounted for 11.6% of global cancer [1]. Due to its indistinct early symptoms, it is often developed at the time of diagnosis. Therefore, the morality rate is very high, and the 5-year overall survival rate is only 15% [2]. Non-small cell lung cancer accounts for more than 80% of total lung cancer, including lung adenocarcinoma (LUAD), lung squamous cell carcinoma (LUSC), and large cell carcinoma (LCLC). Different subtypes have different origin, histological, genetic, and epigenetic changes [3,4]. These differences are closely related to their unique response to treatment [5,6]. Therefore, the study of different subtypes of diagnostic and prognostic markers can help the implementation of precision treatment and improve patient survival rate.

The centromeric protein CEP55, also known as c10orf3 and FLJ10540, is encoded by the CEP55 gene and is widely expressed in different types of tissues, particularly in proliferating tissues [7]. It is located on the centromere of the centrosome and plays an important role in cell mitosis by synergy with CDK1, ERK2 and PLK1 [8]. CEP55 is highly expressed in a variety of cancers, such as breast cancer [9], prostate cancer [10], kidney cancer [11], thyroid cancer [12], and so on. Related clinical studies have shown that CEP55 can be used as a diagnostic and prognostic marker for several cancers. For example, Nina Hauptman et al [13] found that CEP55 is a potential biomarker for colorectal cancer through bioinformatics methods and clinical studies; Yang Jia et al [14] found that in esophageal squamous cell carcinoma (ESCC), CEP55 overexpression is associated with poor prognosis in patients with locally advanced ESCC. Overexpression of CEP55 was significantly associated with a reduction in overall survival after surgery. The 5-year survival rate of patients without CEP55 overexpression was significantly higher than that of patients with CEP55 overexpression. However, there are few studies on whether CEP55 can be used as a marker for LUAD and LUSC diagnosis and prognosis. Therefore, it is meaningful to investigate the diagnostic and prognostic value of CEP55 in LUAD and LUSC.

The present study compared the value of CEP55 as a diagnostic and independent prognostic factor in LUAD and LUSC by bioinformatics analysis. In addition, we also studied the co-expressed genes of CEP55 in LUAD and LUSC, and performed enrichment analysis on these genes to excavate the possible signaling pathways involving CEP55.

## 1 Materials and methods

### 1.1 Analysis of the expression of CEP55 using the cancer genome atlas database and Gene Expression Omnibus database

The gene CEP55 was searched using the data of the TCGA database (http://ualcan.path.uab.edu/cgi-bin/ualcan-res.pl) to analyze the differential expression of CEP55 in some solid tumors and corresponding normal tissues. Data analysis was conducted by Firebrowse (http://firebowse.org/). LUAD chips GSE10072 (n = 104, including 58 tumors and 49 non-tumor tissues, GPL96 [HG-U133A] Affymetrix Human Genome U133A Array) and LUSC chips GSE75037 (n = 166, including 83 cases of lung adenocarcinoma and 83 matching adjacent non-malignant lung tissues, GPL6884 Illumina HumanWG-6 v3.0 expression beadchip) were obtained from Gene Expression Omnibus (GEO) database (https://www.ncbi.nlm.nih.gov/geo/). The differential analysis was carried out using R language “edgeR” package, and normal sample was taken as control. The expression of CEP55 was analyzed compared with normal lung tissues separately, taking |logFC| > 2, adj.pvalue <0.01 as screening criteria of differentially expressed mRNA.

### 1.2 Cancer genomics analysis using cBioPortal

To explore the metabolic pathways involving the CEP55 genes, cBioPortal (http://www.cbioportal.org/) was used to excavate the data of CEP55 in LUAD and LUSC databases. Genes that had similar expression pattern to CEP55 (|Pearson’s r|>0.6, q value < 0.05) were selected as co-expression genes of CEP55. Then conducted GO, KEGG enrichment analysis to these genes.

### 1.3 Statistical analysis

Statistical analysis was performed using SPSS 21.0 statistical software (SPSS, Inc, Chicago, IL, USA). The relationship between CEP55 and clinicopathological features was evaluated by χ2 test. According to whether patients had lung cancer, constructed ROC curve of the expression level of CEP55 to determine its value of diagnostic marker. ROC curve of CEP55 expression level was constructed according to patient’s OS time (Overall Survival) and RFS time (Recurrence-free survival). The expression level of CEP55 was selected when the Yoden index reaches the maximum to determine the value of CEP55 as the prognostic marker. In the meantime, evaluated the difference between survival curves by Log-rank test. COX single factor regression method was used to investigate whether high expression of CEP55 and other clinical factors can be used as independent prognostic factors in patients with LUAD and LUSC; COX multivariate regression was used to explore whether the cross-relationship of prognostic factors is significantly related to prognosis. P < 0.05 means statistically significant.

## 2 Results

### 2.1 The expression of CEP55 was significantly upregulated in both LUAD and LUSC compared with normal cells

**Fig 1:**
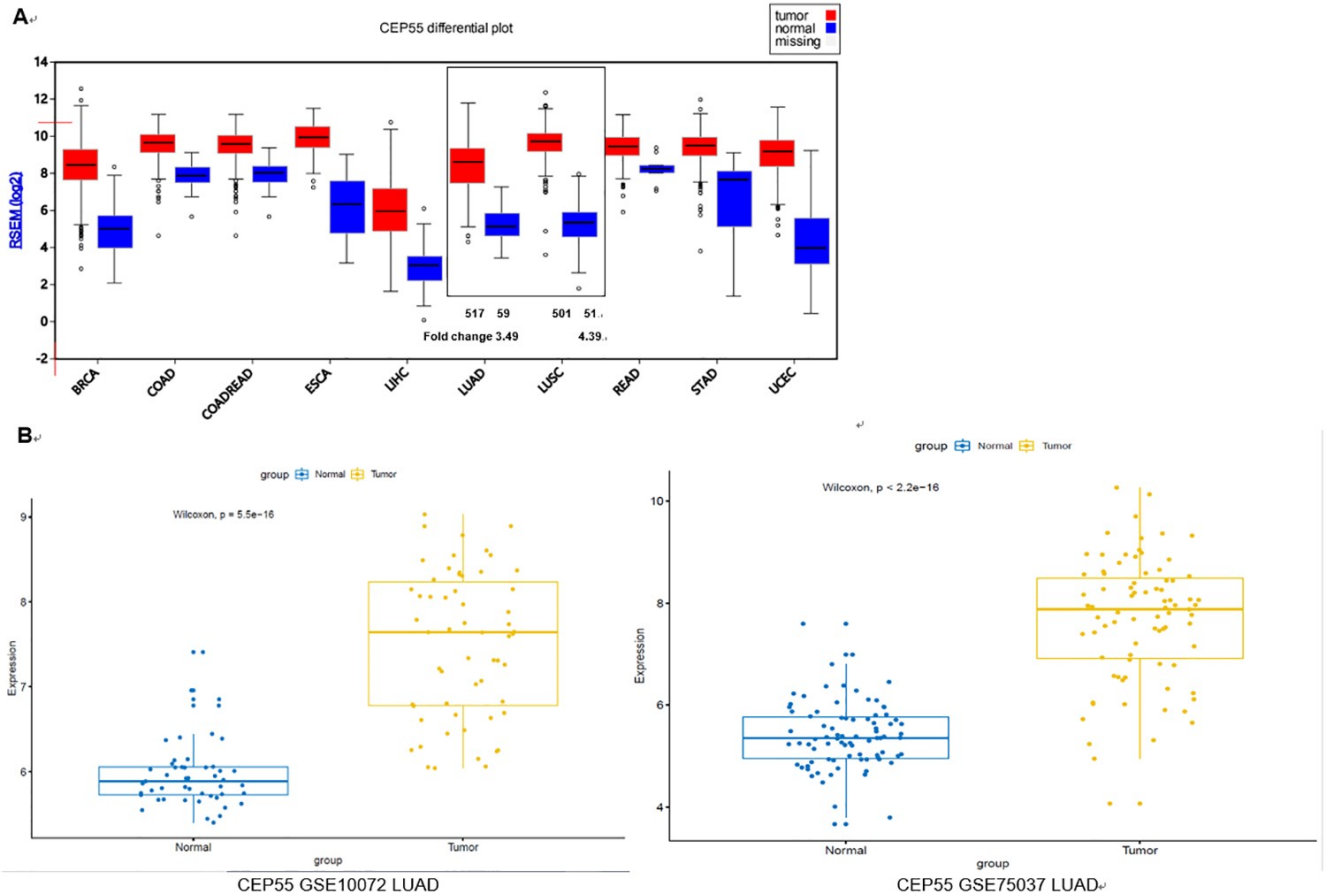
The expression of CEP55 was significantly upregulated in both LUAD and LUSC compared with normal cells. A: CEP55 expression profile was retrieved by TCGA database; B: The expression of CEP55 in LUAD chip of GSE10072 and LUSC chip of GSE75037 was analyzed using the GEO database.

We retrieved some data on the expression of CEP55 in the clinical samples of LUAD and LUSC tissues from the TCGA database. As shown in Fig 1A, the expression of CEP55 was significantly upregulated in both LUAD and LUSC compared with normal cells, with the fold change of which was 3.49 and 4.39, respectively. Meanwhile, we analyzed the expression of CEP55 in LUAD chip of GSE10072 and LUSC chip of GSE75037 using the GEO database. As shown in Fig 1B, the expression of CEP55 was significantly upregulated in both LUAD and LUSC, which was same to the result of TCGA database analysis.

### 2.2 CEP55 could be used as a diagnostic biomarker in both LUAD and LUSC

**Fig 2:**
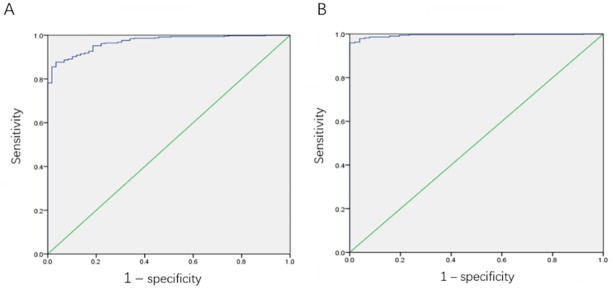
The value of CEP55 as a diagnostic biomarker in LUAD and LUSC. A: ROC curve for evaluating the diagnostic value of CEP55 in LUAD patients, AUC = 0.969; B: ROC curve for evaluating the diagnostic value of CEP55 in LUSC patients, AUC = 0.994.

According to the condition of whether the patient had LUAD or LUSC, ROC curve was constructed to investigate the diagnostic value of CEP55 expression for LUAD and LUSC. For LUAD, the AUC of the curve was 0.969, indicating that the expression of CEP55 could predict whether patients had the risk of LUAD. Take the expression of CEP55 at the highest value of Youden Index as the cutoff value and group the patients. The expression of CEP55 (log2) at this time was 6.725 (Fig 2A).

For LUSC, the AUC of the curve was 0.994, indicating that the expression of CEP55 was of higher diagnostic value for LUSC patients. Take the expression of CEP55 at the highest value of Youden Index as the cutoff value and group the patients. The expression of CEP55 (log2) at this time was 7.977 (Fig 2B). All the results above showed that CEP55 could be used as a potentially diagnostic biomarker for LUAD and LUSC.

### 2.3 The correlation between CEP55 expression and clinicopathological characteristics in LUAD and LUSC patients

We made statistical test for the expression of CEP55 and clinicopathological characteristics (age, gender, smoking history, clinical stage, recurrence and survival status) in LUAD and LUSC patients from the TCGA database, and analyzed their correlation. A total of 494 LUAD patients were analyzed, among which 137 patients existed high expression of CEP55 and 357 patients existed low expression of CEP55. As shown in Table 1, for the patients with LUAD, age, smoking history, clinical stage and survival status were significantly correlated with CEP55 expression (P<0.05), while recurrence was not (P = 0.0629). As shown in Table 2, for the patients with LUSC (N = 504), only age and gender were significantly correlated with CEP55 expression (P<0.05), and the correlation between clinical stage, recurrence and survival status and CEP55 expression was not statistically significant (P>0.05). All the results above showed that the expression of CEP55 could well predict the clinical stage and survival status of LUAD rather than LUSC.

**Table 1:** The association between *CEP55* expression and the demographic and clinicopathological parameters of patients with primary LUAD in TCGA.

Parameters		*CEP55 expression*	*CEP55 expression*	χ^2^	*p* Value
		High (N = 335)	Low (N = 169)		
Age (Mean ± SD)		64.34043 ±10.19204	67.30303 ± 9.12142		0.00171165
Gender	Female	166	105	7.149173	0.007499858
	Male	169	64		
Smoking History	1/2	73	24	4.747795	0.02933586
	3/4/5	189	110		
	Null	73	35		
Clinical Stage	I/II	249	142	7.74474	0.005386923
	III/IV	82	23		
	Discrepancy+null	4	4		
Recurrence status	No	174	103	3.458584	0.06292419
	Yes	109	43		
	Null	52	23		
Living Status	Living	195	127	13.9704	0.000185711
	Dead	140	42		

**Table 2:** The association between *CEP55* expression and the demographic and clinicopathological parameters of patients with primary LUSC in TCGA.

Parameters		*CEP55 expression*	*CEP55 expression*	χ^2^	*p* Value
		High (N = 137)	Low (N = 357)		
Age (Mean ± SD)		64.9403 ± 8.269667	68.09577 ± 8.518631		<0.001
Gender	Female	23	105	8.218	0.004
	Male	114	252		
Smoking History	1/2	22	49	0.353	0.552
	3/4/5	91	240		
	Discrepancy+null	24	68		
Clinical Stage	I/II	109	291	0.277	0.599
	III/IV	27	63		
	Discrepancy+null	1	3		
Recurrence status	No	77	209	0.000222	0.988
	Yes	27	73		
	Null	33	75		
Living Status	Living	84	198	1.384	0.239
	Dead	53	159		

### 2.4 The expression of CEP55 was significantly correlated with the OS and RFS of LUAD rather than LUSC

**Fig 3:**
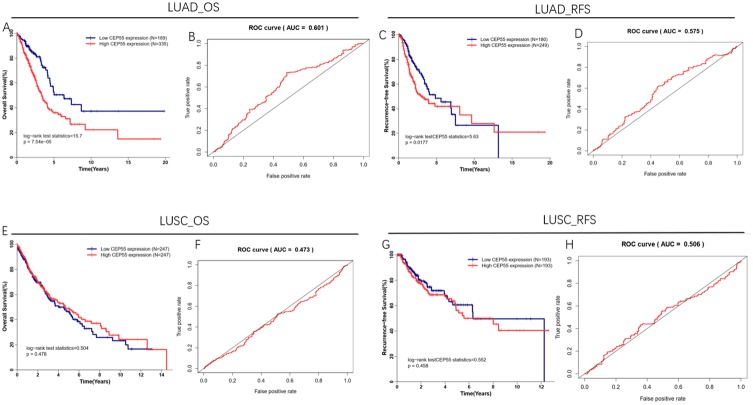
The correlation between CEP55 expression and the OS and RFS of patients. A: The correlation between CEP55 expression and the OS of LUAD patients; B: ROC curve for evaluating OS by CEP55 in LUAD patients; C: The correlation between CEP55 expression and the RFS of LUAD patients; D: ROC curve for evaluating RFS by CEP55 in LUAD patients; E: The correlation between CEP55 expression and the OS of LUSC patients, F: ROC curve for evaluating OS by CEP55 in LUSC patients; G: The correlation between CEP55 expression and the RFS of LUSC patients; H: ROC curve for evaluating RFS by CEP55 in LUSC patients.

Take the expression of CEP55 at the highest value of Youden Index as the optimal cutoff value, and construct ROC curve for CEP55 expression according to the OS time and RFS time of patients. The results showed that in LUAD, the AUC of the ROC curve was 0.601 (Fig 3B) and 0.575 (Fig 3D), respectively, while that in LUSC was 0.473 (Fig 3F) and 0.506 (Fig 3H), respectively, which indicated that CEP55 has better prediction value for LUAD patients. In addition, from the OS curve (Fig 3A) and RFS curve (Fig 3C), we could find that LUAD patients with low CEP55 expression had better prognosis, and their OS and RFS were significantly higher than those of LUAD patients with high CEP55 expression (P<0.05). Also, from the OS curve (Fig 3E) and RFS curve (Fig 3G), we could find that there was no significant difference between the OS and RFS of LUSC patients with high CEP55 expression and low CEP55 expression (P>0.05). All the results above showed that the expression of CEP55 was significantly correlated with the OS and RFS of LUAD rather than LUSC.

### 2.5 CEP55 expression could be used as an independent prognostic factor for LUAD patients rather than LUSC patients

Univariate Cox regression method was used to discuss whether CEP55 expression and other clinical factors (age, gender, smoking history and clinical classification) were correlated with OS and RFS. Factors with statistical significance in univariate analysis were further analyzed using multivariate Cox regression method to determine their role as independent prognostic factors. As shown in Table 3, the OS and RFS of LUAD patients were significantly correlated with clinical classification and CEP55 expression (P<0.05) rather than the age, gender and smoking history (P>0.05), which showed that clinical classification and CEP55 expression could be used as independent prognostic factors for LUAD patients, and high expression of CEP55 could be a predictor of poor prognosis.

**Table 3:** Univariate and multivariate analyses of OS/RFS in patients with primary LUAD.

parameters	Univariate analysis			Multivariate analysis		
	Hazard.Ratio	CI95	P.value	Hazard.Ratio	CI95	P.value
**OS**						
Age(>65 vs < = 65)	1.196	0.89–1.606	0.235			
Female vs Male	0.967	0.722–1.294	0.820			
Smoking history history 2/3/4/5 vs. 1	0.93	0.616–1.404	0.730			
Clinical stage III/IV vs. I/II	2.638	1.933–3.602	<0.001	2.455	1.794–3.36	<0.001
*CEP55* expression High vs. Low	1.983	1.404–2.802	<0.001	1.82	1.28–2.588	0.001
**RFS**						
Age(>65 vs < = 65)	1.305	0.94–1.811	0.112			
Female vs Male	1.1	0.797–1.518	0.562			
Smoking history history 2/3/4/5 vs. 1	1.249	0.778–2.005	0.357			
Clinical stage III/IV vs. I/II	1.702	1.162–2.492	0.006	1.619	1.103–2.376	0.014
*CEP55* expression High vs. Low	1.493	1.07–2.083	0.018	1.454	1.037–2.038	0.030

For LUSC patients, as shown in Table 4, the results of univariate Cox regression analysis indicated that the OS and RFS of patients were only correlated with clinical stage rather than CEP55 expression and other clinical factors (P>0.05), showing that CEP55 could not be an independent prognostic factor for LUSC.

**Table 4:** Univariate analyses of OS/RFS in patients with primary LUSC.

parameters	Univariate analysis		
	Hazard.Ratio	CI95	P.value
**OS**			
Age(>65 vs < = 65)	1.27	0.953–1.692	0.102653
Female vs Male	0.836	0.607–1.152	0.273392
Smoking history history 2/3/4/5 vs. 1	0.588	0.26–1.332	0.203431
Clinical stage III/IV vs. I/II	1.562	1.134–2.152	0.006389
*CEP55* expression High vs. Low	0.814	0.595–1.113	0.197644
**RFS**			
Age(>65 vs < = 65)	0.973	0.649–1.46	0.89667
Female vs Male	0.64	0.395–1.037	0.070149
Smoking history history 2/3/4/5 vs. 1	0.393	0.143–1.078	0.06966
Clinical stage III/IV vs. I/II	1.992	1.237–3.208	0.004581
*CEP55* expression High vs. Low	1.347	0.885–2.049	0.164307

### 2.6 Analysis on related signaling pathways and co-expression genes of CEP55 in LUAD and LUSC

**Fig 4:**
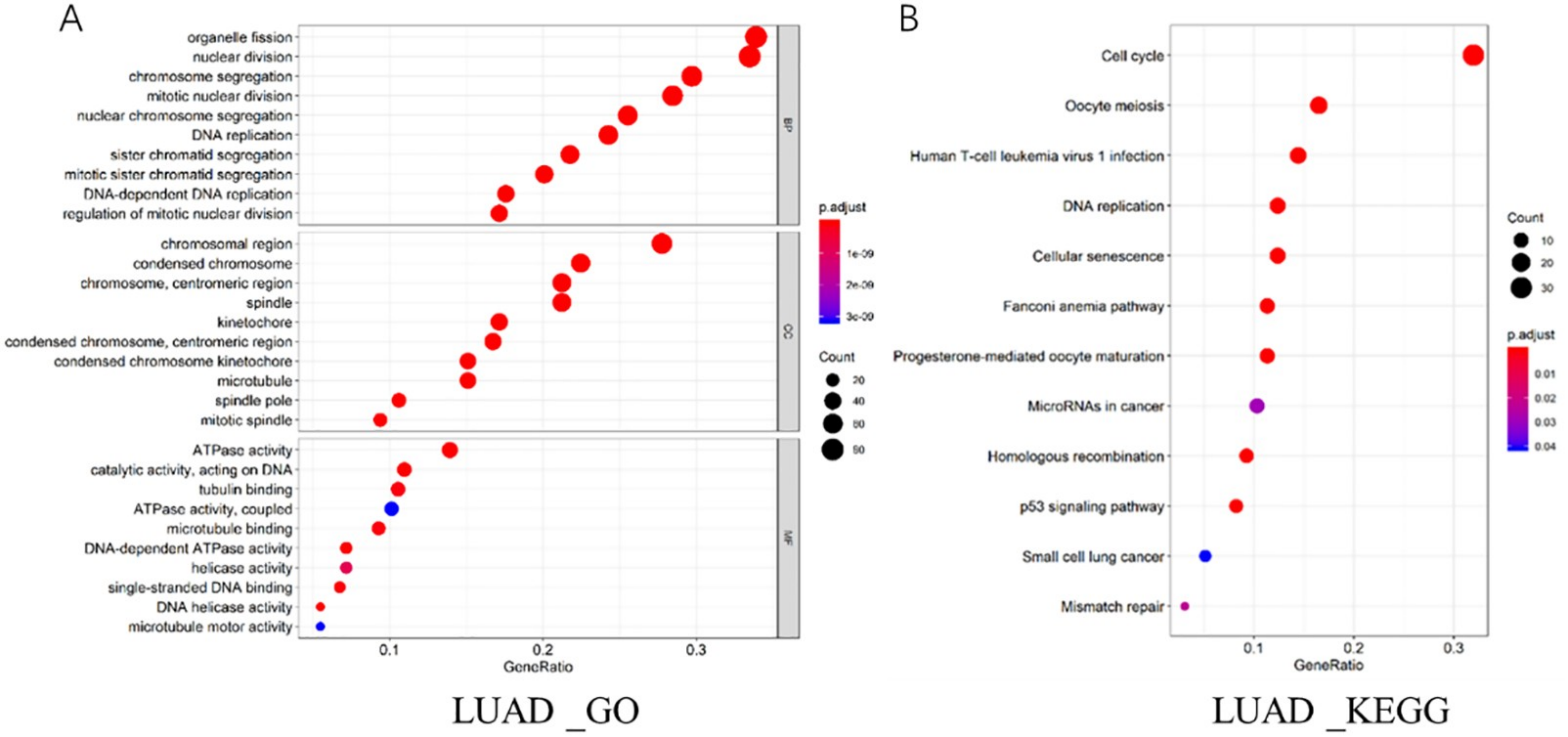
Analysis on related signaling pathways and co-expression genes of CEP55 in LUAD.

Co-expression genes that had similar expression pattern to CEP55 (|Pearson’s r|>0.6, qvalue < 0.05) were excavated by cBioPortal in LUAD and LUSC databases, finding that CEP55 co-expressed with 253 genes in LUAD. To further study the signaling pathways that CEP55 might be involved in, the co-expression genes of CEP55 in LUAD were enriched and analyzed by Go (Fig 4A) and KEGG (Fig 4B), finding that these co-expression genes were correlated with cell cycle, DNA replication and P53 signaling pathway.

However, only two co-expression genes of CEP55 (|Pearson’s r|>0.6, qvalue < 0.05), KIF11 and KIF20B, were excavated in LUSC database.

## 3 Discussion

Most lung cancer patients are diagnosed at terminal stage. In the early stage of treatment, lung cancer would metastasize and develop drug resistance. Therefore, the clinical effect of lung cancer remains ineffective. Different lung cancer subtypes show different clinical features and prognosis. It is important to study the diagnostic and prognostic markers of two different types of NSCLC, LUAD and LUSD. In this study, we found CEP55 was highly expressed in both LUAD and LUSC compared to normal cells, and was more significantly expressed in LUSC. Previously, CEP55 has proven to be a useful biomarker for a variety of cancers, including lung cancer [15,16]. However, its predictive value in the two subtypes of LUAD and LUSC in non-small cell lung cancer has not been reported. By constructing the ROC curve, we found that CEP55 can be used as a diagnostic marker for LUAD and LUSC, and people with high expression of CEP55 have a higher risk of cancer.

CEP55 is a 55 KD-sized centrosome-associated protein that plays an important role in membrane fission events and is a key regulator of cell division [17,18], and is closely related to the development of cancer. Studies have shown that high expression of CEP55 can promote cancer proliferation, migration and invasion [19–21]. As an oncogene, up-regulation of CEP55 is also associated with poor prognosis in certain cancers. Chao Jiang [22] et al. have found that the expression of CEP55 was significantly increased in non-small cell lung cancer tissues, and overexpression of CEP55 was associated with poor prognosis in patients with NSCLC. But in this study, the AUC value of the ROC curve for assessing the prognostic value of CEP55 expression to NSCLC was 0.600. Therefore, this study refines the NSCLC classification and constructs a ROC curve to evaluate the prognostic value of CEP55 expression for LUAD and LUSC. It is found that the ROC curve AUC of LUAD is 0.628, which is higher than that reported in the literature, while the LUC curve of LUAC is less than 0.5 with no predictive value. At the same time, we analyzed the OS and RFS survival curves and found that the correlation between CEP55 expression and LUAD patients was statistically significant. LUAD patients with high expression of CEP55 showed shorter OS and RFS time. However, this rule was not found in LUSC patients. Same results were also obtained by COX regression analysis of the correlation of CEP55 expression with patient OS and RFS. Above results indicate that CEP55 has a potential value as an independent prognostic factor for LUAD, but has a poor predictive value for LUSC.

Exploring the differences in the molecular mechanisms of CEP55 in LUAD and LUSC is beneficial to further explore the new NSCLC targeted diagnostic approach, which is helpful for the effectiveness of anticancer therapy, thereby improving the overall survival rate of lung adenocarcinoma of lung cancer. Several studies have found that CEP55 is involved in the phosphatidylinositol-3-kinase (PI3K)/Akt signaling pathway by triggering Akt phosphorylation [23–25]. According to reports in the literature, CEP55 binds and interacts with PI3KCA (p110), which may activate AktS473 to promote transcription of downstream target genes [19]. However, in LUAD and LUSC, the specific regulatory mechanisms and differences are still unclear. In present study, we used cBioPortal to co-express CEP55 gene in LUAD and LUSC databases finding that LUAD and LUSC are quite different. CEP55 has a total of 253 co-expressed genes in LUAD. The KEGG and GO enrichment analysis of these genes revealed that they are involved in various signal pathways such as cell cycle, DNA replication and P53 pathway. These signaling pathways are closely related to the development of cancer. Further study of the role of CEP55 in LUAD is of great significance. In LUSC, we only screened two co-expressing genes for CEP55, namely KIF11 and KIF20B.

In conclusion, the present study analyzed the value of CEP55 as a diagnostic and independent prognostic factor for LUAD and LUSC by biomarker. CEP55 was found to be a diagnostic biomarker for both NSCLC subtypes. However, when CEP55 was selected as a prognostic factor for NSCLC, high expression of CEP55 was more for LUAD than LUSC patients. Therefore, the prognostic value of CEP55 gene expression in LUAD patients is higher than that of LUSC patients, which will be of important clinical significance.

## Supporting information

S1 Data (TXT)

S2 Data (TXT)

S3 Data (TXT)

S4 Data (TXT)

S5 Data (TXT)

S6 Data (TXT)

S7 Data (TXT)

S8 Data (TXT)
